# Knowledge and perceptions of type 2 diabetes among Ghanaian migrants in three European countries and Ghanaians in rural and urban Ghana: The RODAM qualitative study

**DOI:** 10.1371/journal.pone.0214501

**Published:** 2019-04-02

**Authors:** Ama de-Graft Aikins, Francis Dodoo, Raphael Baffour Awuah, Ellis Owusu-Dabo, Juliet Addo, Mary Nicolaou, Erik Beune, Frank P. Mockenhaupt, Ina Danquah, Silver Bahendeka, Karlijn Meeks, Kirstin Klipstein-Grobusch, Ernest Afrifa-Anane, Liam Smeeth, Karien Stronks, Charles Agyemang

**Affiliations:** 1 Regional Institute for Population Studies, University of Ghana, Legon, Ghana; 2 Kumasi Centre for Collaborative Research, Kwame Nkrumah University of Science and Technology, Kumasi, Ghana; 3 Department of Non-Communicable Disease Epidemiology, London School of Hygiene and Tropical Medicine, London, United Kingdom; 4 Department of Public Health, Academic Medical Center, University of Amsterdam, Amsterdam, the Netherlands; 5 Institute of Tropical Medicine and International Health, Charite, University Medicine Berlin, Berlin, Germany; 6 Department of Molecular Epidemiology, German Institute of Human Nutrition, Potsdam-Rehbruecke, Nuthetal, Germany; 7 MKPGMS-Uganda Matyrs University, Kampala, Uganda; 8 Julius Global Health, Julius Center for Health Sciences and Primary Care, University, Medical Center Utrecht, Utrecht, the Netherlands; Middlesex University, UNITED KINGDOM

## Abstract

African migrants in Europe and continental Africans are disproportionately affected by type 2 diabetes (T2D). Both groups develop T2D at a younger age, and have higher morbidity and mortality from T2D and complications, compared to European populations. To reduce risk, and avoidable disability and premature deaths, culturally congruent and context specific interventions are required. This study aimed to: (a) assess perceptions and knowledge of T2D among Ghanaian migrants in Europe and their compatriots in Ghana and (b) identify specific perceptions and knowledge gaps that might predispose migrants to higher risk of diabetes. Data was gathered through 26 focus groups with 180 individuals, aged 21 to 70, from Amsterdam, Berlin and London and rural and urban Ashanti Region, Ghana. Thematic analysis of the data was informed by Social Representations Theory, which focuses on the sources, content and functions of social knowledge. Three key insights emerged from analysis. First, there was general awareness, across migrant and non-migrant groups, of T2D as a serious chronic condition with life threatening complications, and some knowledge of biomedical strategies to prevent diabetes (e.g healthy eating) and diabetes complications (e.g medication adherence). However, knowledge of T2D prevention and reduction of diabetes complications was not comprehensive. Secondly, knowledge of biomedical diabetes theories and interventions co-existed with theories about psychosocial and supernatural causes of diabetes and the efficacy of herbal and faith-based treatment of diabetes. Finally, migrants’ knowledge was informed by both Ghanaian and European systems of T2D knowledge suggesting enculturation dynamics. We discuss the development of culturally congruent and context-specific T2D interventions for the research communities.

## Introduction

Sub-Saharan African origin populations in Europe are disproportionately affected by type 2 diabetes (hereafter T2D) compared with the host European populations. Similarly, the prevalence rates of T2D have increased in many sub-Saharan African (SSA) countries. African migrants in Europe develop T2D at a younger age, and have higher morbidity and mortality from T2D and complications, compared to European populations [1]. In Africa, an estimated 14.2 million people live with diabetes (3.2% of the regional population of 441million), with almost three times as many having impaired glucose tolerance [2]. It is estimated that two-thirds of populations at risk are undiagnosed or unaware they have diabetes [2].

There has been a concerted effort by global and regional policymakers to address chronic non-communicable disease (NCD) prevention and control in order to reduce levels of risk within the lay population and to prevent avoidable complications and premature death within populations living with the major NCDs, including diabetes. There is a general consensus that effective NCD prevention and control strategies require empowering patients and communities to understand and address specific conditions [3].

In this paper, we report a qualitative study that examined knowledge and perceptions of diabetes among Ghanaian migrants in three European cities—Amsterdam, Berlin and London—and Ghanaians in rural and urban Ashanti region of Ghana. The qualitative study was a component of the RODAM (**R**esearch on **O**besity and **D**iabetes among **A**frican **M**igrants) study, a large scale multi-centre cross-sectional study with over 6000 respondents, which aimed to examine the complex interplay between environmental exposures (e.g. lifestyle, psychosocial stress), culture (e.g beliefs, habits) and genetics and to identify specific risk factors to guide effective intervention programmes in the stated study communities [1,4]. In Ghana, participants were recruited from rural and urban Ashanti region because previous studies including our pilot study in Amsterdam showed that the majority of prospective study participants in Europe originated from this region of Ghana [5]. Because a key aim of the broader study was to study gene-environmental interactions, it was necessary to get as similar a population as possible across the study sites.

The qualitative component was nested within the main study, with the majority of participants recruited from the main study cohort, and it focused on the environmental and cultural study questions.

The aims of the qualitative study were:

1. To assess perceptions and knowledge of T2D among Ghanaian migrants in Europe, and their compatriots in Ghana (including gaps in perceptions and knowledge that might raise the risk of T2D); and
2. To examine how insights on perceptions and knowledge could be used to develop appropriate T2D interventions for these communities.

This paper reports analysis of data gathered through focus groups with 180 lay healthy individuals across the five study sites, on their knowledge and perceptions of T2D.

## Background and conceptual framework

A number of studies have been conducted on knowledge and perceptions of T2D in selected African countries [6–12]. Studies have focused on perspectives from people living with T2D, caregivers and lay healthy individuals. This body of work has reported awareness of T2D in all study communities. The majority of study communities demonstrate some knowledge of the risk factors of T2D, with accuracy of knowledge associated with education, social class and urban residence. The studies also show that individuals subscribe to multiple causal theories of diabetes, blending biomedical, common sense and supernatural theories [6–8]. While individuals tend to privilege biomedical theories and interventions over theories and interventions of competing health systems (such as ethnomedicine and faith healing), biomedical prevention and treatment strategies can be undermined by a number of factors including poor access to formal healthcare services, poverty and health disabling beliefs [6, 8–10].

Research on knowledge and perceptions of T2D among African migrant communities in Europe is very limited [13,14]. However emerging work on lay beliefs of hypertension, chronic disease and general illness in African migrant communities suggest that migration and acculturation (Box 1) play important roles in migrants’ perspectives on chronic conditions [15–19].

**Box 1. Acculturation: definition and categories**

Acculturation is defined as “changes that take place as a result of contact with culturally dissimilar people, groups, and social influences” and is typically studied in individuals living in countries or regions other than where they were born, e.g among immigrants and refugees [20]. Four acculturation categories have been identified: (1) assimilation (adoption of receiving culture and rejection of heritage culture); (2) separation (rejection of receiving culture and retention of heritage culture); (3) integration or biculturalism (adoption of receiving culture and retention of heritage culture); (4) and marginalization (rejection of both the heritage and receiving cultures). Recent elaborations of the concept point to the complex and multidimensional nature of the phenomenon, in terms of how migrants, as collectives, sub-groups or individuals draw on one or more of the four main strategies depending on specific interactional situations (e.g at work, on the street, in familiar multicultural gatherings). Enculturation, for example, is a nuanced version of the integration/bicultural category and refers to “the process of selectively acquiring or retaining elements of one’s heritage culture while also selectively acquiring some elements from the receiving cultural context”. These multidimensional categories present equally nuanced implications on associations between acculturation and psychological and mental health, with biculturalism and enculturation associated with the most positive psychosocial outcomes [20].

For example, African migrants in the Netherlands and the UK understand the common risk factors of hypertension, but the content of their knowledge is shaped by enculturation processes [15–17]. They subscribe to common sense and supernatural causal theories of hypertension in similar ways to African communities–they draw on their heritage cultures (Box 1). However, aspects of their understandings of hypertension are shaped by prevailing ideas about health, illness and chronic conditions in their host countries–they also draw on their receiving cultures (Box 1). The mix of established cultural ideas from their home countries and new ideas from their host countries shape eclectic treatment strategies across a range of health systems within their host and home countries [18].

The value of the RODAM qualitative study lies in the goal of systematically and critically comparing perceptions and knowledge between migrant and non-migrant Ghanaian groups and, within the context of migrant experiences, identifying which aspects of perceptions and knowledge are drawn from the Ghanaian cultural setting and which aspects are drawn from their new European mainstream cultural contexts. Identifying patterns of convergence and divergence in perceptions and knowledge will inform the development of appropriate context-specific and culturally nuanced interventions for both migrant and non-migrant groups.

This study used Social Representations Theory (hereafter SRT), a social psychological theory, as a broad conceptual framework to examine T2D knowledge and perceptions. The theory focuses on the production of everyday practical social knowledge and facilitates an examination of the sources, contents and functions of social knowledge [21–23]. One of its key strengths is its focus on the dynamic and contradictory structure of everyday knowledge. ‘Cognitive polyphasia’, a central concept of the theory is defined as the state in which different kinds of knowledge, with inherently different or contradictory rationalities, coexist in the minds of the same individual or group [23,24]. Cognitive polyphasia, for example, captures the way African communities may attribute T2D or hypertension simultaneously to biomedical and supernatural causes.

In this study we applied SRT to examine the content, sources, functions of knowledge of diabetes across the study sites and to examine the gaps in knowledge by contrasting the content of lay knowledge with the existing research and expert knowledge on T2D. We were particularly interested in the complex nature of T2D attributions, as reported in aforementioned studies. We anticipated that understanding these dimensions of knowledge and perceptions would inform the development of culturally appropriate and context-specific interventions. Therefore, we applied the concept of ‘cognitive polyphasia’ to examine competing rationalities shaping diabetes knowledge and perceptions. We also examined the sources of knowledge in order to identify how sociocultural processes shaped the production of knowledge, including enculturation processes for migrant Ghanaians.

## Methods

### Study areas

The full details of the RODAM Study including rationale, conceptual framework and methodology have been described elsewhere [1,4]. The majority of the qualitative study participants were recruited from the broader RODAM study. At the conceptual stage of the study, the RODAM team agreed on a segmentation approach for the FGDs which would target lay healthy individuals, comprising groups of young men and women (aged 35 years and below) and groups of older men and women (aged above 35 years). The aim of FGDs was to facilitate discussion, examine social ideas about T2D, and to examine these ideas at the intersection of gender, age and migration status. In Europe, FGDs were conducted with Ghanaian migrants living in various neighbourhoods in Amsterdam, London and Berlin. In Ghana, focus group discussions were conducted in three urban communities and two rural communities in the Ashanti region. The urban communities were Asuoyeboah, (a suburb in Kumasi, the capital town of the region), Atonsu, and Obuasi. The rural communities were Denyase, and Akwaaduo. Three separate field teams were trained to conduct FGDs, with standardised pilot-tested FGD guides, in Ghana, London and Amsterdam/Berlin, under the supervision of the first author (across all sites), the fifth author (in London) and the last author (in Amsterdam). The teams were given access to the list of RODAM participants in each site. We contacted eligible participants by phone (across all sites) and through community gatekeepers (in rural Ghana) to request their participation in the qualitative study and arranged FGD meetings in community centres. We also employed snowballing techniques to reach eligible individuals who did not participate in the broader RODAM study. We aimed to convene 4–6 FGDs each within each site and to recruit between 160 and 240 individuals. While we oversampled in rural and urban Ghana, and recruited 6 groups in Berlin, we faced practical challenges in achieving full participation in London and Amsterdam. It was relatively easy to recruit participants in urban and rural Ghana because participants were bound by geographical location and, in rural Ghana, fairly similar work routines. For example, in rural Ghana, FGD meetings were scheduled for when community members returned from the farm and this ensured full participation. In the European cities, participants lived in disparate parts of the cities, with various work commitments–this meant that while potential participants expressed interest they could not participate in the FGDs for practical reasons. We applied alternative recruitment approaches, such as organizing mixed gender groups across the European sites and targeting strong groups with shared social backgrounds such as friendship groups and church members. We had greater recruitment success with the migrant communities, when we employed individual interviews scheduled at participants’ preferred times and locations (for other aspects of the broader qualitative study to be reported elsewhere).

### Participants’ profiles

Twenty-six (26) focus group discussions were conducted across the five study locations, between 2012 and 2014. Six (6) group discussions were conducted in Berlin, two (2) each conducted in Amsterdam and London and eight (8) each in urban and rural Ashanti region. A total of 180 individuals were involved in the group discussions. Groups were segmented into older males, older females (≥35 years), younger males, younger females (<35 years) and mixed gender groups (in the European cities). Table 1 presents the demographic details of the FGD participants.

**Table 1:** Demographic details of FGD participants.

Characteristic	Total N = 180	Rural Ghana* n = 64	Urban Ghana** n = 50	Amsterdam n = 14	London n = 12	Berlin n = 40
***Sex***						
Male	86	33	26	11	4	12
Female	94	31	24	3	8	28
***Age***						
≤35	90	29	38	1	12	10
>36	90	35	12	13	-	30
***Education***						
No education	14	14	-	-	-	-
Primary	17	13	3	1	-	-
Middle/JHS	42	26	9	7	-	-
Secondary	28	6	11	1	-	10
Higher	33	5	1	5	12	10
No response	46	-	26	-		20
***Religion***						
No Religion	9	9	-	-	-	-
Christian	130	55	24	9	12	30
No response	41	-	26	5	-	10
***Ethnicity***						
Akan	121	64	24	5	8	20
Ga	17	-	-	7	4	6
Ewe	2	-	-	2	-	-
No response	40	-	26	-	-	14

*FGDs were conducted two rural communities–Denyase and Akwaaduo

**FGDs were conducted three urban communities–Obuasi, Atonsu, and Asuoyeboah.

The FGDs covered five broad themes presented in Box 2. Discussion on T2D formed part of a longer discussion on T2D, obesity and general health and illness beliefs. The FGD guide covering T2D questions is provided in the appendix (S1 File). Group discussions across the five sites lasted between 1 hour and 2 hours.

**Box 2. Summary of FGD themes on diabetes**

1. Knowledge on T2D (knowledge on nature of condition)
2. Causes of T2D (poor lifestyles, overweight/obesity, witchcraft and/or sorcery etc.)
3. Treatment of T2D (biomedical treatment, herbal medication, spiritual treatment etc.)
4. Complications of T2D (knowledge of complications)
5. Perceptions of T2D (ideas around illness disclosure)
6. Source of T2D and health knowledge/information

Ethical approval of the study protocols was obtained for all sites from the respective ethics committees in Ghana (School of Medical Sciences/Komfo Anokye Teaching Hospital Committee on Human Research, Publication & Ethical Review Board), the Netherlands (Institutional Review Board of the AMC, University of Amsterdam), Germany (Ethics Committee of Charite-Universitätsmedizin Berlin) and the UK (London School of Hygiene and Tropical Medicine Research Ethics Committee) before data collection began in each country [4]. Informed written consent was obtained before interviews and FGDs were conducted.

### Data analysis

All the FGDs were conducted, transcribed and quality-checked, before analysis was conducted by a team of trained analysts. FGDs conducted in local languages (Twi mainly, Ga to a minor extent) were transcribed directly into English by a team of transcribers with Ga, Twi and English language competence. The transcripts went through two further rounds of quality checks: first by a designated language quality checker with English, Ga and Twi language skills (Author 3), and then through group discussion with field interviewers led by the first author. Coding and analysis was informed by a thematic analysis approach [25] and facilitated by the qualitative software package, Atlas.ti.

Coding was done deductively and inductively. Deductive codes were derived from research insights of existing diabetes qualitative studies conducted in Ghana, African countries and migrant communities in Europe [5,7,26,27]. Inductive codes were derived from original narratives and ideas expressed by the groups linked to specific contexts across the five sites. Previous research, which generated the deductive codes, showed that causal theories of diabetes included sugar, poor diets, contaminated foods, the supernatural, physiological disruption and family history. Our coding process began with identification of these reported causal theories as our deductive codes. Once we had exhausted active identification of deductive codes we read the transcripts closely to identify inductive codes. At this stage we were interested in identifying original theories of diabetes as well as new variations on existing causal theories. The coding frame developed through an iterative process of multiple group discussions and critical attention to consensus, conflict and absence across the group narratives.

The second stage of analysis involved a more nuanced linkage between codes, themes, appropriate quotes, existing empirical data, study objectives and the conceptual framework. Two considerations shaped interpretive analysis of codes and themes. First, the content of the lay causal theories and how they differed across study sites (Study Objective 1); and second, their similarities to, and differences from established medical theories of diabetes causation and complications in order to identify gaps in knowledge (Study Objective 1).

Finally, to identify complex patterns in causal attributions that would facilitate culturally appropriate and context specific interventions (Study Objective 2), we examined the categories of knowledge shaping representations of diabetes. For example, inductive and deductive coding yielded eight lay causal theories of T2D. In order of dominance the causal theories of diabetes were: unhealthy food/dietary practice, family history, supernatural causes, overweight/obesity, psychological stress, smoking, alcohol consumption, physical inactivity and contaminated foods. We conceptualised these as natural, supernatural, social, psychological and structural theories. At stage 2 of analysis we identified four sources of these theories of diabetes: pluralistic health systems, religious institutions, mass/social media and family/social networks. Mapping content and sources of knowledge facilitated the identification of four categories of knowledge: scientific (biomedical), cultural, common sense and religious. Box 3 outlines the key elements of each category of knowledge.

**Box 3. Characteristics of the four categories of knowledge**

**Cultural knowledge**

Core beliefs about supernatural causes of misfortune and crisis. This category of knowledge was drawn from traditional and cultural sources (e.g. family, herbalists) and included themes on witchcraft, sorcery and spiritual action. Witchcraft has been defined as malevolent action caused by people (witches) who possess mystical powers that are used to harmful ends. Sorcery has been defined as malevolent action caused by ordinary people who do not possess mystical powers but have knowledge of and access to spells or rituals, that are used to harmful ends. Some themes were buttressed by religious (Christian) knowledge on the supernatural, the divine and fate. This category of knowledge informed supernatural, social and psychosocial theories of T2D and T2D complications.

**Common sense knowledge**

Characterized by social observations and everyday knowledge in the public sphere, including the media (mass and social). This category of knowledge was the most dynamic as it included mixed themes from all the remaining categories of knowledge. It is also in the reporting of common sense knowledge that enculturation processes became most salient for migrant groups. This category of knowledge informed all the theories of T2D and T2D complications–natural, supernatural, social, psychosocial, psychological, structural—but also influenced social critique of these theories.

**Religious knowledge**

Characterised largely by Christian doctrine on faith, God’s grace and the healing power of prayer. This category of knowledge was drawn from the Christian religious sphere. It shaped ideas and reinforced cultural beliefs about spiritual and supernatural forces such as the power of the devil or Satan to cause diseases including diabetes through direct action or indirect action (via sorcery and witchcraft). This category of knowledge informed supernatural, social and psychosocial theories of T2D and T2D complications.

**Scientific knowledge**

Encompassed ideas derived largely from biomedicine about causes and complications of diabetes and other health conditions. This category of knowledge was drawn from medical sources (clinics, hospitals, medical/health websites) and public health education in the social sphere (e.g churches, billboards, websites) and included themes such as heredity, obesity and poor diets as causes of diabetes. There was a strong correlation between education, proximity to a significant other with T2D and sophistication of biomedical ideas. This category of knowledge informed natural theories of T2D and T2D complications.

## Results

The results are presented under three sections: (I) Knowledge and perceptions of T2D and T2D complications; (II) Sources of T2D knowledge and perceptions; and (III) Functions of T2D knowledge. For each section we present the full range of themes, starting with dominant and consensual themes and ending with outlying themes expressed by fewer groups. In presenting quotes our central aim is to present ‘*units of meaning*’ that capture the essence of narratives and arguments [28]. When a theme is consensual across the groups, one quote that best captures shared meaning is used as illustration. When there are variations of consensual themes a range of quotes will be presented to reflect the variation in views. When results focus on conflict, a range of quotes that capture/illustrate this conflict will be presented. Box 4 presents the key for identifying the sources of selected quotes.

**Box 4. FGD labels**

RG–Rural Ghana; UG—Urban Ghana; A–Amsterdam; B–Berlin; L–London

OMG—Older male group; YMG–younger male group; OFG–Older female group;

YFG–younger female group; MGG–Mixed gender group

### 1. Content of knowledge and perceptions on T2D

All the participants across the five study sites had heard of T2D. T2D was understood to be a chronic condition and was associated with sugar and sugary foods. It is important to note that T2D is associated with sugar/sugary foods in the Ghanaian public imagination because the condition is referred to as ‘sugar disease’ in a number of local Ghanaian languages, including Twi (*esikyere yare*), the language spoken by the majority of study participants. However, most participants presented multiple causal theories for the condition, which have been categorised as natural, supernatural, psychosocial, and structural.

#### Natural causes of T2D

Natural causal theories of diabetes emerged from fourteen groups across the study locations. Participants mentioned family history of diabetes as a possible cause of diabetes. The common explanation was that if an older generation member of one’s family (nuclear or extended) had diabetes, later generations of that family were likely to have the condition. Other participants observed that people were born with the condition, without specifying family history. Some participants had a family history of diabetes–they provided details of family members who lived with or had died from diabetes.

It could be hereditary. If your father has diabetes, you can have that condition. [UG -YMG]

I don’t really know much about diabetes. But what I know is my grandfather had it and he passed it on to most of his children. Most of them died out of diabetes. Two years ago his daughter’s legs were amputated. My father also died shortly after we got to know he is diabetic. He fell sick for just a week. After series of tests the doctor informed me it is diabetes. Before he died he lost his sight. The doctor confirmed it was caused by diabetes. [B-MGG]

A second natural cause of T2D was overweight and obesity. This was mentioned by ten groups across the five study locations.

Yes! obesity makes one prone to diabetes. [A-MGG]

Fatness can also lead to diabetes. [RG-OFG]

In broader discussions on overweight and obesity, there was consensus across all groups that ‘fatness’ arose from natural, social and psychological factors. Natural factors included heredity, ethnicity, and unique ‘God-given’ physiology.

#### Supernatural causes of T2D

Fifteen groups across all the study sites associated T2D with supernatural causes. The causes included: witchcraft, sorcery (or bought disease), the devil, and general spiritual action (through nonspecific evil forces and dark spirits). Participants’ definitions of witchcraft and sorcery were aligned with anthropological definitions of both phenomena (see Box 3). However, some participants believed sorcery and witchcraft could operate simultaneously for the same condition. Others placed the root cause of sorcery and witchcraft within the realm of higher powers beyond malevolent individuals, such as the devil, Satan and generalised spiritual powers. These eclectic reasoning processes blended cultural and religious understandings of misfortune.

Someone can buy diabetes for you spiritually. [UG -YFG]

Diabetes is a disease and for diseases like malaria spiritual powers can buy it for you. [UG -OMG]

Diabetes can be bought and passed down to you through witchcraft especially if Satan hates you. [UG -OMG]

Sometimes we hear people say ‘family sickness’, but what that actually means is that a member of the family has bought that sickness for the entire family and its passed on to you if you let down your guard. So, satanic sicknesses do exist. [RG -OMG]

A second set of participants believed T2D was caused by a mix of spiritual and natural factors: this group drew on Christian doctrine in their dual theory of T2D.

I think (it) is difficult to look at them separately. There may be a disease or illness that you contract through natural causes but is aggravated through spiritual causes or you can’t ignore the, spiritual cause, you shouldn’t, at least as a Christian that is what we believe, we shouldn’t ignore the spiritual dimension in the same way we shouldn’t be ignoring the natural means of going to see a doctor and taking medication [L-MGG]

I personally wouldn’t feel comfortable living with relatives with this belief. I need someone to completely understand that the two need to work in together and it is also the power of God that can bring healing to that person, if is the will of God. I think is very dangerous to say that this is solely a spiritual cause or this is solely a natural cause because is far far more complex than that [L-MGG]

A third set of participants did not personally associate T2D with supernatural factors. This group recognised that supernatural causal theories of chronic and mysterious illnesses were common in Ghanaian and African cultures, but did not share—or expressed scepticism at—the perceived collective belief that diabetes was spiritually caused.

Most of the diseases that kill us are the faults of these pastors so I really like what you are doing. When you get to these pastors talk to them… instead of advising the person to go to the hospital these pastors put the blame on your grandmother (*alluding here to witchcraft*). [B-MGG]

**Interviewer:** can diabetes be bought spiritually for someone? **Respondent:** …I have not seen some before, it is all rumour, is even said AIDS can be bought for someone [RG-OMG]

#### Psychosocial causes of T2D

Psychosocial causes of T2D hinged on socially mediated behavioural and lifestyle factors, everyday habits shaped by socio-economic status, and negative psychological states. All the groups across the five sites mentioned aspects of psychosocial causes.

Socially mediated behavioural factors included, in order of dominance: unhealthy dietary practices, physical inactivity, alcohol overconsumption, and smoking. Most of the groups across the five study sites attributed diabetes to unhealthy eating practices. Some participants focused on the consumption of unhealthy foods, including sugary foods, fatty foods and fast or junk foods. Other participants focused on unhealthy dietary practices including overeating, eating late and sleeping immediately after late night eating.

Your eating habits can also cause diabetes [B-OFG]

If you take in sugar and a lot of sweets, you can get it (diabetes). [RG—OMG]

Late night eating, and sleeping as soon as you eat can cause diabetes [RG -YMG]

T2D was attributed to physical inactivity by groups across all five study locations. In broader discussions on health and illness, participants defined physical activity as exertion of the physical body through work or various forms of exercises.

Lack of physical exercise can be a cause of diabetes [A-OMG]

T2D was attributed to alcohol overconsumption by nine groups across the five sites. While a general observation was made on alcohol overconsumption, participants did not elaborate on types and quantities of alcohol implicated in diabetes risk. A few participants, in the Berlin migrant groups, disputed the association between alcohol overconsumption and diabetes.

The drinks we take give us diabetes. I drank a few bottles of alcoholic drinks on Friday, Saturday and Sunday and I had to go see the doctor, my blood sample was taken and a few days later the hospital called my house and told me to come to the hospital because my sugar level has risen. [B-MGG]

Drinking cannot get you diagnosed with diabetes. Drinking may alter your thinking abilities but can’t give you diabetes. I am not a doctor though but I have also not heard a doctor say alcohol causes diabetes [B-MGG]

Three groups in rural and urban Ghana, and London attributed diabetes to cigarette smoking. Similar to the observations on alcohol consumption, no elaborations were made on types and quantities of cigarettes.

Also, smoking cigarette causes the disease (diabetes) [RG—OFG]

Everyday habits shaped by socio-economic status related to common perceptions that associated T2D and other common chronic conditions to wealth. Two groups in rural Ghana and Berlin associated T2D with wealth (*esikafo yare*, in Twi). Participants observed that wealthy individuals and groups had greater access to unhealthy foods (processed, fatty, sugary foods) and therefore had a higher risk of getting T2D.

I have heard that it’s (diabetes) a disease for the rich [RG-YFG]

Two psychological causal theories of diabetes emerged in five groups across the five sites. Five groups (in urban and rural Ghana and Berlin) attributed diabetes to (too much) thinking. Four groups (in urban Ghana, Amsterdam, London and Berlin) attributed diabetes to psychosocial stress arising from family tensions and livelihood struggles.

Another prominent cause (of diabetes) is deep thinking [B-OFG]

Stress also causes diabetes. [B-MGG]

#### Structural causes of T2D

Three structural causes of diabetes were highlighted: contaminated foods, working conditions and medical technologies. Contaminated foods and family planning methods have been characterised as structural factors because both themes were viewed as problems arising from external institutions: the agricultural sector for the former, and the health sector for the latter.

Four groups (in rural and urban Ghana, Amsterdam and Berlin) associated diabetes with contaminated staples. These groups expressed a shared perception that there was a rising use of toxic agro-chemicals in production, harvesting and storage of staple food crops and they believed these practices compromised the nutritional quality and safety of these foods.

For me I can say it’s (diabetes) caused by the food we have being eating of late. You see, of late even garden eggs is grown with fertilizer, as well as tomatoes, even cassava and plantain are planted with fertilizer. [RG-YFG]

There was a general sense, expressed by a number of the groups in the European cities, that working conditions in these cities increased psychosocial stress and prevented the adoption of healthy lifestyles among Ghanaian communities. Two kinds of poor working conditions were reported: work demanding long hours, and engagement in multiple jobs. Work-related stresses were associated with a variety of prevalent chronic conditions, including T2D, within the migrant communities.

Mental illness is not common here to me, but rather stress and depression. That state of being is a very serious situation and can cause someone to be severely sick. Due to the problems surrounding work here, quite a number of people have this problem. [A-MGG]

One group (in rural Ghana) attributed T2D to medical technologies in reproductive health services.

I think family planning pills and injections can also cause diabetes [RG-OMG]

### Content of knowledge on T2D complications

Some groups across the five sites presented natural and psychosocial theories for T2D complications. Group narratives often conflated types of complications, causes of complications and consequences of complications.

### Natural causes of T2D complications

Participants outlined six types of natural T2D complications: foot complications (slow healing wounds, leg amputations), eye problems, sexual dysfunction, other chronic conditions (hypertension, heart problems, stroke, kidney failure) and general physical disorder and weakness.

Foot complications: The most common complication mentioned by the groups related to foot damage, or what experts refer to as ‘diabetes foot’. Thirteen groups across four sites (rural and urban Ghana, Amsterdam and Berlin) mentioned slow healing wounds or sores. Ten groups across three sites (rural and urban Ghana and Berlin) mentioned foot amputations. Eight groups across four sites (rural and urban Ghana, Amsterdam and Berlin) mentioned painful swelling of the feet.

If care is not taken and you cut yourself, it turns into a chronic sore. This can lead to your limbs being amputated. [B-MGG]

Sexual dysfunction: Eight groups across four sites (in rural Ghana, urban Ghana, Amsterdam and Berlin) mentioned sexual dysfunction as a complication of diabetes. Sexual dysfunction was perceived in terms of loss of libido arising from the stresses of living with diabetes and impotence as a side effect of diabetes medication. Gender differences were not highlighted.

I know that diabetes can also affect the sexual life of a person and make an individual impotent. [A-OMG]

Eye problems: Eye problems were mentioned by six groups (in rural and urban Ghana, Berlin and London) as a complication of diabetes. Participants observed that diabetes could result in difficulties in seeing or complete loss of sight.

It (*diabetes*) can cause blindness [B-MGG]

Other chronic conditions: Four groups in Berlin and London observed that diabetes could lead to hypertension, heart problems and heart attacks. Groups in rural and urban Ghana mentioned stroke as a complication. Individuals in three groups in Amsterdam, Berlin and London mentioned kidney failure as a complication.

Diabetes often comes with high blood pressure [B-MGG]

One can also get stroke [UG-OFG]

Interviewer: What are the complications of diabetes …? Respondent1: Is kidney failure part of it? …That is the only thing I know, kidney failure. [L-MGG]

Physical disorder and weakness: A mix of complications were outlined that can be placed under the broad category of physical disorder and weakness. This included frequent urination (8 groups in rural Ghana, urban Ghana, Amsterdam and Berlin), loss of physical strength associated with frequent urination (same groups as previous), dental hygiene problems (2 groups in urban Ghana and Amsterdam) and risk of premature death (5 groups in rural and urban Ghana and Berlin).

#### Psychosocial outcomes of T2D

Eight groups (in rural and urban Ghana, Berlin and London) mentioned psychosocial stress and financial distress as outcomes of living with T2D.

You can’t sleep when you go to bed, you keep thinking [RG-OFG]

You will also not have money [RG-OFG]

In Table 2 below, we provide a summary of the spread and dominance of causal theories for T2D and T2D complications to highlight similarities and differences in knowledge and perceptions in rural and urban Ghana and in the European cities.

**Table 2:** Spread and dominance of theories of T2D and T2Dcomplications across the five study sites.

	Rural Ghana (8FGDs)	Urban Ghana (8 FGDs)	Amsterdam (2 FGDs)	Berlin (6 FGDs)	London (2 FGDs)
**Diabetes**					
Natural					
*Heredity*	**+**	**+++**	**+++**	**+++**	**+++**
*Overweight/obesity*	**+**	**+**	+++	**+++**	**+++**
Supernatural	**+++**	**+++**	**+++**	**+++**	**+++**
Psychosocial					
*Poor Dietary practices*	**+++**	**+++**	+++	**+++**	**+++**
*Physical inactivity*	**+**	**+**	+++	**+++**	**+++**
*Alcohol*	**+**	**+**	+++	**+**	+++
*Smoking*	**+**	**+**	o	o	+++
*Wealth status*					
*Too much thinking*	**+**	**+**	+	**+**	+++
*Psychosocial stress*	**+**	**+**	+	**+++**	+++
Structural	**+**	**+**	+	+++	+++
**Diabetes complications**					
Natural					
*Foot problems*	**+++**	**+++**	**+++**	**+++**	+++
*Eye problems*	**+**	**+**	o	+	**+++**
*Other chronic conditions*	**+**	**+**	+++	**+++**	+++
*Physical disorder*	**+**	**+**	+++	**+++**	**+++**
Psychosocial					
*Psychosocial stress*	**+**	**+**	o	**+**	**+++**
*Financial distress*	**+**	**+**	o	**+**	**+++**

**Notes: +++:** theme mentioned in at least half of groups in one site

+: theme mentioned in a minority of groups (less than half); o: theme not mentioned in any group in target site

### II. Sources of T2D knowledge

Four main sources of knowledge on diabetes and diabetes complications were identified: pluralistic health systems, religious institutions, media (mass and social) and family and social networks. It is important to note that the four causal theories outlined in the previous section–natural, supernatural, psychosocial, structural–were drawn from eclectically from these sources. For example, some FGD participants were educated on medical aspects of T2D by nurses during church outreach services.

#### Pluralistic health systems

The most common source of diabetes knowledge was pluralistic health systems. In order of numerical dominance, participants accessed knowledge from medical doctors (14 groups), nurses (8 groups), hospitals (during formal consultation) (8 groups) and herbalists (7 groups) and dieticians (2 groups). Among the rural and urban Ghanaian groups, reference to herbalists was limited to the Ghanaian traditional medicine sector. Among the migrant Ghanaian groups, reference to herbalists included Ghanaian traditional medicine as well as complementary and alternative medicine sectors such as Chinese medicine.

You will get such information from your (medical) doctor. [B-MGG]

Advertisement from the herbalists who claim they can cure diseases such as these (diabetes) [RG-OMG]

I get such information from hospitals [RG-YFG]

#### Religious institutions

Churches were important sources of diabetes and other health-related knowledge across all sites. Participants from nine groups mentioned churches and church groups as their source of knowledge. Participants from seven groups mentioned religious leaders as their source of information. Other participants referred to gaining knowledge in churches through public health education provided by health professionals as part of church outreach.

The groupings in the churches like say, the women’s group [RG-YMG]

They allow two nurses to come to our church and teach us especially on pressure concerning the food you have to eat and so many things [UG-OFG]

**Respondent**: I really like that church we went to that they have their own like GP’s, surgery room thing…with erm everything ECG machine everything. **Interviewer**: Is it a Ghanaian church? **Respondent1**: Yes [L-MGG]

#### Media

Some groups accessed health information from traditional visual and print media: TV, radio, newspapers, magazines and booklets. Others mentioned the internet and social media as their source of knowledge. Ghanaians in London, Amsterdam and Berlin were more likely to access information from the internet. Younger Ghanaians in London accessed social media (Facebook, Instagram, blogs and apps), as well as public health messages from billboards and posters in public places and on public transport (e.g. buses, trains).

We also get information from the television and they heighten the awareness of these diseases. [UG-OMG]

The internet is used most of the time. [B-MGG]

A lot of people focus on it like nowadays like healthy eating and like exercise and a lot of

people now on this like been in this fitness training do you see a lot of this on Instagram. [L-MGG]

#### Family, friends and social networks

Family, friends and other social networks constituted a final source of information on diabetes and general health conditions. Participants from eight groups mentioned family members as their source. Participants from eight groups mentioned friends as their source of knowledge and participants from six groups mentioned social groups as their source of knowledge. Social groups included people living with diabetes.

When you contact the disease and you discuss with your friend, your friend can educate you [UG-OFG]

Some participants, in both migrant and non-migrant groups, raised concerns about the legitimacy of health information drawn from social networks. Others suggested that people with diabetes or other chronic conditions did not disclose their conditions to their friends or social networks for fear of gossip and social stigma.

One might confide in a friend but the friend would go round selling the problems so it discourages people from sharing their problems. [B-MGG]

Table 3 presents a summary of the sources of knowledge on T2D and T2D complications and their relative dominance across the five sites.

**Table 3:** Spread and dominance of sources of T2D knowledge across the five study sites.

	Rural Ghana (8FGDs)	Urban Ghana (8 FGDs)	Amsterdam (2 FGDs)	Berlin (6 FGDs)	London (2 FGDs)
**Pluralistic health systems**					
*Hospitals*	**+**	**+**	**+++**	**+**	**+**
*Doctors*	**+++**	**+++**	**o**	**+++**	**+++**
*Nurses*	**+**	**+**	o	**+++**	**o**
*Herbalists (Ghanaian)*	**+**	**+**	**+**	**+**	**+**
*Herbalists (Asian)*	**o**	**o**	**o**	**o**	**+**
**Religious institutions**					
*Churches*	**+**	**+++**	+++	**+**	+++
*Church groups*	**+**	**+**	+	+	+
**Family and social networks**					
*Family members*	**+**	**+++**	+++	**+**	+++
*Friends*	**+**	**+++**	+++	+	+++
*Social groups*	**+**	**+**	+	+	+
**Media**					
*Print media*	**o**	**+**	**+++**	**+++**	+++
*Visual/audio media*	**+++**	**+++**	**+++**	**+++**	+++
*Internet/Social media*	**o**	**+**	+++	+++	**+++**

**Notes: +++:** theme mentioned in at least half of groups in one site

+: theme mentioned in a minority of groups (less than half); o: theme not mentioned in any group in target site

### III. Functions of T2D knowledge

The concept of ‘functions of diabetes knowledge’ was operationalised in terms of what participants reported doing with their knowledge of diabetes and diabetes complications. For example, did an understanding of the risk factors for diabetes inform healthier lifestyle practices for them or did an understanding of diabetes complications inform the advice they provided to family members or friends living with diabetes?

Three themes emerged from the group discussions on the subject of reducing the risk of diabetes complications. The first theme was medication management. Participants from ten groups across the five study locations indicated that biomedical/pharmaceutical medicines were required to reduce the risk of diabetes complications. Within the same groups, participants also stated that herbal medicines could be used to reduce the risk of diabetes and diabetes complications, in combination with biomedical treatment or in isolation. There was conflict on this theme, with some group members dismissing the efficacy and safety of herbal medicines.

One can prevent diabetes by receiving medical treatment from doctors in a hospital [RG-OFG]

Herbal medicine is very effective in treating diabetes, when you take the western medicine in excess you can get other complications [B(3)-MGG]

I went to Greenwich to visit my sister and there was this Asian shop like a Chinese shop that they do all this herbal stuff like they have this sort of Chinese medication that people drink. People are really buying it. [L-MGG]

Many people in Ghana have died because they use herbs and go to pastors instead of seeking proper health care [B[2]-MGG]

The second theme was diet management. The majority of groups across the five study sites mentioned eating foods low in sugar and starch as a strategy for preventing diabetes and diabetes complications. One group in Amsterdam included consumption of low salt foods as a diet management strategy.

Once we know what the causes are, we just have to avoid them. Like avoiding sugary foods. [A-MGG]

The third theme was faith-based coping. Participants from eight groups (five in rural and urban Ghana, one each in Amsterdam, London and Berlin) advocated the healing power of prayer. They observed that regular and committed prayer could reduce the risk of diabetes complications. Some participants associated the healing power of prayer with spiritually caused diabetes and suggested that prayer could cure diabetes if one had strong faith. Others suggested that prayer worked in combination with dedicated biomedical treatment. This argument followed the assertion that diabetes arose from complex natural and spiritual factors (see section I).

There are testimonies, people having headaches and they prayed and the headache is gone. That is how we build our faith, when you start small with these things. [L-MGG]

I know prayer can cure diabetes; it all depends on the faith you have [B-MGG]

In Ghana we believe some diseases can be given to you spiritually, if the disease is given to you spiritually then a true pastor can pray for the person to get cured but even then, you will still have to go to the hospital before you can be cured completely [B-MGG(3)]

A number of participants discussed diabetes treatment and management through the lens of healer shopping (across biomedicine, ethnomedicine and religious systems), and dual/multiple use of pluralistic treatment methods. Some advocated healer shopping and multiple treatment strategies. Others emphasised the superior efficacy of biomedicine over other treatment methods. Among the Berlin groups, there were explicit within-group disagreements on these issues.

It depends on which one you want to adopt because, biomedical drugs can treat it likewise some researched and certified herbs. God also works in mysterious ways so you can be healed by prayers just that it will be based on your faith [B[5]-MAG]

Many people in Ghana have died because they use herbs and go to pastors instead of seeking proper health care [B[2]-MAG]

Table 4 summarises the spread and dominance of functions of T2D knowledge.

**Table 4:** Spread and dominance of ‘functions of T2D knowledge’ across the five study sites.

	Rural Ghana (8FGDs)	Urban Ghana (8 FGDs)	Amsterdam (2 FGDs)	Berlin (6 FGDs)	London (2 FGDs)
**Medication management**					
*Pharmaceutical medication adherence*	**+**	**+**	**+++**	**+++**	**+++**
*Herbal treatment*	**+**	**+**	**+++**	**+**	**+++**
**Diet management**					
*Low sugar and starch foods*	**+++**	**+++**	+++	**+++**	+++
*Low salt foods*	o	o	+	o	o
**Physical activity**	**o**	**o**	+++	**o**	0
**Faith-based management**					
*Prayer/faith*	**+**	**+**	+++	+	+++
**Healershopping**	**+**	**+**	+	+	**+**

**Notes: +++:** theme mentioned in at least half of groups in one site

+: theme mentioned in a minority of groups (less than half); o: theme not mentioned in any group in target site

## Discussion

This study aimed to: (a) assess perceptions and knowledge of diabetes among Ghanaian migrants in Europe and their compatriots in Ghana and (b) examine how insights from identified perceptions and knowledge could be used to develop appropriate T2D interventions. We will first discuss the study findings in relation to these study aims and discuss implications for primary prevention.

### Perceptions and knowledge of T2D among Ghanaian migrants in Europe and their compatriots in Ghana

There was comprehensive awareness of T2D as a chronic condition across all study sites and a consensual association of diabetes with ‘sugar’ and ‘sugary foods’. T2D was also attributed to multiple causes beyond sugar/sugary foods. Four causal theories were identified: supernatural (sorcery, witchcraft, Satan, evil spirits), natural (heredity, overweight/obesity), psychosocial (behavioural and lifestyle factors including poor dietary habits and practices, physical inactivity, alcohol overconsumption, smoking, psychological factors including thinking, psychosocial stress), and structural (contaminated foods, harmful working conditions, family planning methods). These four causal theories have been reported in studies on diabetes in Ghana [8,26,29], and in other African countries among people with diabetes and lay healthy individuals [5,6,10–12,30]. Respondents drew interchangeably from the four causal theories in similar ways to reports in existing studies.

However, some important differences emerged in this study. First, participants made stronger and more nuanced associations between psychosocial factors and diabetes compared to narratives in existing African/Ghanaian studies. In previous studies associations are made between individual (or intrapersonal) level psychological factors—in particular thinking too much and worry–and diabetes risk. In this study, participants highlighted intrapersonal (as above), interpersonal, social and structural level psychosocial factors in T2D risk and diabetes management: specifically, stressful family and social relationships, the double-edged nature of community and social support, and poor working conditions. Secondly, in this study and previous studies, T2D knowledge was drawn eclectically from a range of sources spanning family spaces to the mass media. In this study the public sphere sources of diabetes and health knowledge were broader in migrant narratives compared to rural/urban Ghanaian narratives and sources reported in existing Ghanaian/African studies. This difference was clearly associated with the role of social media and the structure of public health education in the European cities, where health information is provided on multiple platforms, from GP offices to messages on buses and trains (see Table 3). This expanded source of health information shaped differences in diabetes perceptions and knowledge between Ghanaian migrants and urban/rural Ghanaians.

A clear pattern emerged on differences in knowledge between study locations, which could be linked to two sets of representations of diabetes. The first set of representations could be referred to as core representations. Core representations were shaped by established cultural and religious knowledge and focused on supernatural causal theories and associated common sense theories rooted in traditional belief systems. These were relatively stable representations tied to Ghanaian heritage culture. They highlighted the salience of cultural and religious identity in the production of knowledge about health and illness among Ghanaians at home and abroad (Box 3 and Table 2).

**Fig 1:**
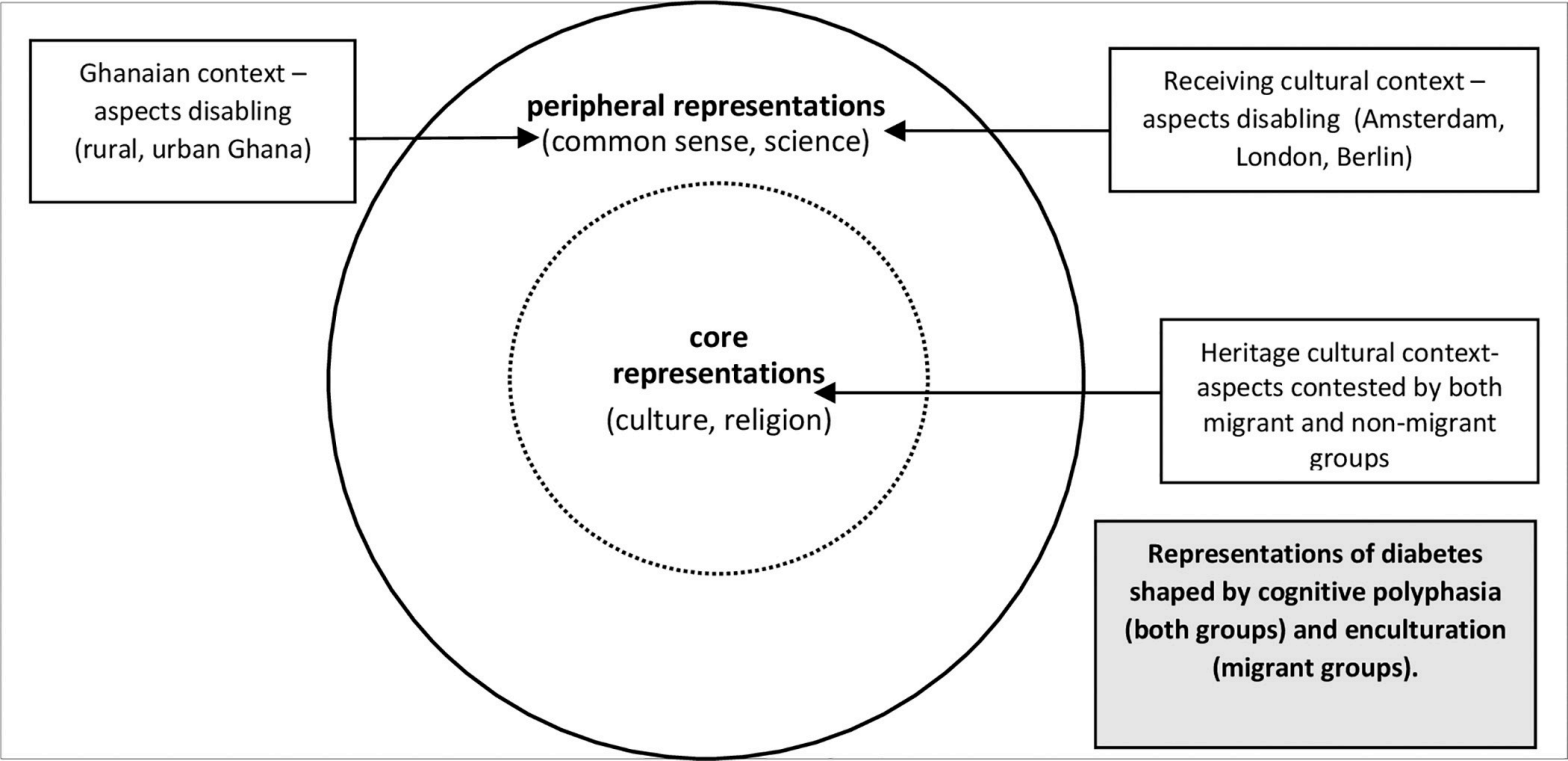
Core and peripheral representations of T2D among Ghanaian migrants in Europe and Ghanaians in Ghana.

The second set of representations can be described as peripheral representations. These were shaped by scientific knowledge and common sense knowledge. The quality of scientific knowledge was shaped by access to comprehensive biomedical information–here migrant groups by their accounts had greater access to stronger health systems and public health education. The quality of common sense knowledge was shaped by access to credible community and media sources, and to some extent people with T2D. Since common sense knowledge was tied to dynamic social contexts, peripheral social representations demonstrated a strong relationship between the quality of T2D information in the social/public context and the quality of social knowledge held by individuals and groups. In the case of migrant groups, peripheral representations were more comprehensive in content and clearly rooted in biomedical, public health and social messages tied to their receiving cultural context. This suggested that enculturation processes (see Box 1) played an important role in the social representations of T2D among Ghanaian migrants. This was similar to studies on African migrant knowledge on hypertension [15,17]. Fig 1 presents a visual relationship between core representations and peripheral representations in the narratives of migrant and non-migrant Ghanaians.

### Perception and knowledge gaps that might predispose Ghanaian migrants to higher risk of T2D and implications for developing appropriate T2D interventions

Perception and knowledge gaps–and misconceptions–emerged across the five causal theories that can predispose Ghanaian migrant (and non-migrant) communities to a higher risk of diabetes. For some groups, there were gaps in knowledge on aspects of diabetes risk (e.g. smoking, alcohol overconsumption, ageing) and diabetes complications (e.g. eye problems). For other groups established risk factors for T2D and T2D complications were contested (e.g alcohol overconsumption, medical non-adherence, healershopping, dual use). While migrant groups who presented perspectives on diabetes risk and complications had fewer knowledge gaps, not all groups offered their perspectives (Table 2), and some contested established biomedical theories. Limited or poor knowledge about the full range of diabetes risks and complications can have an impact of lifestyle choices and practices and inappropriate management strategies.

A major misconception about diabetes was its attribution to supernatural causes. The supernatural theme was a culturally derived causal theory that had no rational convergence with biomedical theories. The core ideas embedded in these theories have been reported in the African literature on chronic illness beliefs and are reported to shape heath and illness behaviour in complex ways. Previous diabetes studies in Ghana and other African countries suggest that the expression of a supernatural causal theory of diabetes does not necessarily lead individuals to seek supernatural interventions from traditional religious shrines or Christian and Islamic faith healers [7,31]. The relationship between belief and practice is complex, because supernatural beliefs often co-exist with other causal theories which have stronger empirical salience (eg poor eating habits) and well-tested treatments (e.g biomedicine). However, for people living with diabetes, supernatural causal theories can influence healthcare practices negatively particularly when individuals lack access to quality biomedical care (diagnoses, affordable treatment, means to measure blood glucose levels). Similar results emerged in this study. First, belief in supernatural causes did not lead to advocacy of traditional religious interventions (e.g shrine priests); but to Christian faith-based healing and prayer. Second, there was evidence of ideological struggles with supernatural causal theories within both migrant and non-migrant groups, as well as the functions of these theories. Migrant groups engaged in more critical debates about the legitimacy of these theories, but T2D knowledge in both groups was cognitive polyphasic and migrant groups were influenced by enculturation processes in their representations of diabetes. These ideological struggles suggested a weakening of established core representations and the possibility for developing education interventions that demystify and deconstruct strongly held cultural beliefs and practices.

Finally, there were two important themes emerging in group narratives that required consideration in diabetes interventions.

The structural theme included ‘contaminated foods’ and poor working conditions. The association between contaminated foods and diabetes and other NCDs has been reported in the literature on lay NCD beliefs among native Canadians [32], Latino communities [33] and among Ghanaians, Nigerians and African migrants in the UK [17,34]. This association is not salient in the expert medical literature, but appears in the literature on the nexus between agriculture, food security and public health nutrition [35]. This suggests the need for a synthesis of ideas across these expert fields. Both migrant and non-migrant groups associated T2D with psychosocial stressors in family and social life. Migrant groups also associated psychosocial stressors with poor working conditions. Studies suggest that migrant groups are more likely to end up in 3D working conditions: dirty, dangerous and demeaning [1]. The salience of multi-level psychological attributions across the study sites and dominance in the migrant communities (both for diabetes risk and diabetes complications) suggest that diabetes interventions for this community should prioritise psychosocial stressors across family, social and working domains.

## Conclusions

This study had a major limitation: we did not get the planned number of participants in London and Amsterdam due chiefly to structural challenges. Aspects of the challenges we experienced have been reported in the literature on accessing minority ethnic participants in social and public health research [36]. We did not achieve meaning saturation with migrant groups and reported insights should therefore be generalised with caution.

However, the challenges experienced during participant recruitment for FGDs shaped creative responses–such as targeting ‘strong groups’ of friends or church members—that present methodological insights for future studies with similar migrant communities. The challenges also emphasised the importance of using mixed qualitative methods in maximising access to, and engagement with, research participants.

More broadly, by employing a theory-driven, multi-site, multi-experiential qualitative approach, the study has identified patterns of T2D perceptions and knowledge across Ghanaian and European locations and age groups that can inform appropriate interventions. This study supports insights from previous studies and adds new insights to the literature. First, our research participants like other African and African migrant communities draw on eclectic theories and sources of information in making sense of T2D. Secondly, while there is general awareness of the public health importance of T2D, complications arising from T2D, and strategies for reducing risk and complications, knowledge is not comprehensive and some misperceptions and misunderstandings exist. In terms of new insights, the study shows that psychosocial theories are multi-level and nuanced and core cultural theories are challenged by some community members. We also focus on an understudied community–migrant Ghanaians in Europe–and present a systematic examination of how their knowledge of T2D is shaped by enculturation processes. This supports the development of interventions that blend existing public health strategies targeting majority European populations with specific culturally congruent adjustments for minority ethnic groups.

Education and lifestyle interventions must emphasise the themes that are already known to lay communities: these include poor diets, alcohol overconsumption, smoking, physical inactivity, overweight/obesity and psychosocial stress. Education strategies must also demystify supernatural causal theories of diabetes, without delegitimizing productive aspects of religious responses to illness. For example, prayer provides important, and empirically proven, spiritual and psychosocial benefits to religious individuals [37]. Public health education is more likely to be successful if accessible and culturally congruent social channels are used for dissemination. In both rural and urban Ghana and the European cities, our empirical data suggests that collaboration between formal healthcare systems and faith-based organizations can create credible and potentially cost-effective sources of disseminating T2D information, screening and providing advice and support for T2D care. Messaging through T2D advocacy groups is also likely to have a positive impact as people with T2D are a credible source of information for lay individuals. In the European cities, health websites and social media health platforms can serve as important channels for diabetes education, particularly among younger Ghanaians.

## Supporting information

S1 File RODAM qualitative study–diabetes FGD guide– 03Feb2019. (DOCX)
