# Supplementary material for: Knowledge and perceptions of type 2 diabetes among Ghanaian migrants in three European countries and Ghanaians in rural and urban Ghana: The RODAM qualitative study
Source: PLoS One. 2019 Apr 2;14(4):e0214501. doi: 10.1371/journal.pone.0214501 (PMC6445464; doi:10.1371/journal.pone.0214501)
Supplement: S1 File — (DOCX) [file pone.0214501.s001.docx]

**RODAM QUALITATIVE STUDY**

**DIABETES FOCUS GROUP GUIDE (PROTOCOL B)**

*Introduction to study/Ethical issues/Preliminary questions on socio-demographic profiles.*

B.1. Have you ever heard about diabetes?

Probe: what kind of condition diabetes is: infectious? chronic?

B.2. Do you know anybody who has diabetes?

B.3. What do you think are causes of diabetes?

Probe: sugar, or sugary food; contaminated foods (e.g. toxic agro chemicals on fruits and vegetables); poor lifestyles (e.g. drinking, smoking); heredity/family history; insulin irregularities; lack of physical exercise; overweight/obesity; pregnancy; witchcraft and/or sorcery; other.

B.4. How can diabetes be treated?

Probe:  Biomedical treatment; list known health centres, medicines and cost

Herbal medication; list known health centres, medicines and cost

Spiritual treatment: (church, traditional religious leader); list known approaches e.g. fasting, dietary restrictions and cost

Home and/or traditional remedies; list known remedies and cost

Probe: whether diabetes can be cured.

B.5 What are the complications of diabetes?

Probe: knowledge of complications by asking respondents to list at least three complications such as eye problems; foot problems; teeth/gum problems; blood pressure/heart problems

B.6. What ideas do people have about diabetes in this community?

Probe: ideas around illness disclosure. Is diabetes a condition that will be disclosed?

B.7. Can diabetes be prevented?

Probe: knowledge of minimising major risk factors: diet; overweight/obesity; smoking; alcohol over consumption

B.8. Apart from diabetes, are there any chronic diseases you know of?

Probe: as many individuals to mention chronic conditions they know.

Probe: Facilitator to mention conditions from the long list after the initial generation of spontaneous responses: **hypertension, cancers, stroke, heart disease, asthma, sickle cell disease, arthritis, epilepsy, mental illness**.

Probe: whether knowledge is based on whether respondents know individuals with these conditions.

B.9. On the scale of 1 to 10, which is the most serious? (**Explain that 1 is least serious (e.g having that condition will not change your life or your identity or cause you to be fearful or worried) and 10 is most serious (e.g the condition can change your life and identity significantly, for example in terms of stigma and/or premature death, and can cause prolonged fear and anxiety**)

B.10. Where do you get your information about health and illness?

Probe: Friends, family, media, healthcare providers, church, mosque, social groups (women’s fellowship, men’s fellowship), other.
